# Quantitative Polymerase Chain Reaction System for Alongshan Virus Detection

**DOI:** 10.3390/mps6050079

**Published:** 2023-09-04

**Authors:** Alexander G. Litov, Egor V. Okhezin, Ivan S. Kholodilov, Alexandra E. Polienko, Galina G. Karganova

**Affiliations:** 1 Laboratory of Biology of Arboviruses, FSASI “Chumakov Federal Scientific Center for Research and Development of Immune-and-Biological Products of RAS” (Institute of Poliomyelitis), 108819 Moscow, Russia; oe-74@mail.ru (E.V.O.); ivan-kholodilov@bk.ru (I.S.K.); polienko.ae@yandex.ru (A.E.P.); karganova@bk.ru (G.G.K.); 2 Department of Biology, Lomonosov Moscow State University, 119234 Moscow, Russia

**Keywords:** Jingmenvirus group, Alongshan virus, qPCR, *Orthoflavivirus*, Yanggou tick virus, tick-borne viruses

## Abstract

The recently discovered Jingmenvirus group includes viruses with a segmented genome, RNA of a positive polarity, and several proteins with distant homology to the proteins of the members of the genus *Orthoflavivirus.* Some Jingmenvirus group members, namely the Alongshan virus (ALSV) and Jingmen tick virus, are reported to be tick-borne human pathogens that can cause a wide variety of symptoms. The ALSV is widely distributed in Eurasia, yet no reliable assay that can detect it exists. We describe a qPCR system for ALSV detection. Our data showed that this system can detect as little as 10^4^ copies of the ALSV in a sample. The system showed no amplification of the common tick-borne viruses circulating in Eurasia, i.e., the Yanggou tick virus—which is another Jingmenvirus group member—or some known members of the genus *Orthoflavivirus*. The qPCR system was tested and had no nonspecific signal for the *Ixodes ricinus, I. persulcatus*, *Dermacentor reticulatus, D. marginatus*, *Haemaphysalis concinna*, and *H. japonica* ticks. The qPCR system had no nonspecific signal for human and sheep serum as well. Overall, the qPCR system described here can be used for reliable and quantitative ALSV detection.

## 1. Introduction

Viruses of the genus *Orthoflavivirus* (family *Flaviviridae*) are small, enveloped, and have a nonsegmented single-strand RNA genome of a positive polarity. The genome encodes a polyprotein that is co- and post-translationally cleaved by viral and cellular proteases into ten proteins [1]. Many orthoflaviviruses are arthropod-borne viruses (arboviruses) and are transmitted by mosquitoes and ticks [1]. Some members of the genus *Orthoflavivirus* are well-known human pathogens, such as the West Nile virus [2], dengue virus [3], Zika virus [4], and tick-borne encephalitis virus (TBEV) [5].

Recently, the number of newly described viruses has increased dramatically, with several novel virus groups being discovered [6,7,8]. The Jingmenviruses (JMV) group is one of these novel virus groups. The genome of the JMV group members consists of four segments. The first segment encodes a protein with homology to the genus *Orthoflavivirus* polymerase [9]. The third segment encodes a orthoflavivirus-like helicase [9,10]. The second segment encodes two-to-three open reading frames with little homology to the *Orthoflavivirus* envelope protein [9,11]. The fourth segment encodes two open reading frames [9].

The JMV members include the Jingmen tick virus (JMTV) [9], Yanggou tick virus (YGTV) [12], Guaico Culex virus [13], Alongshan virus (ALSV) [14,15], and several other viruses [16,17]. The geographic distribution of the JMV group is very wide, encompassing Europe, Asia, America, and Africa [9,12,13,14,15,18,19,20]. JMV group members are considered arboviruses. At least two members of the JMV group, the JMTV and ALSV, are considered tick-borne human pathogens [15,21], and little information on the pathogenicity of other JMV group members exists.

The JMTV was discovered in China and was found to be highly prevalent in tick samples from Hubei province. Moreover, the results of a screening of cattle sera that was conducted using an immunofluorescence assay and RT-PCR revealed that samples from Hubei and Zhejiang, China, were JMTV positive [9]. The results of a follow-up study revealed four human cases in China, which were detected via the high-throughput sequencing of skin biopsies and blood samples. Eight more JMTV cases in humans were retrospectively identified in the same study. All the patients had a history of tick bites, and three of them were coinfected with *Rickettsia* [21]. The reported manifestations of the JMTV infection were fever, headache, malaise (which is typical for febrile illnesses), an itch or painful eschar after the tick bite, and lymphadenopathy [21]. Since the discovery of the JMTV, it has been detected in Europe, Kenya, Japan, and Brazil [22].

The ALSV was also discovered in China during surveillance for tick-borne diseases in a patient with a febrile illness [15]. The results of a follow-up epidemiological investigation in China confirmed the detection of ALSV in 86 patients. Most patients (95%) had a clear history of tick bites before disease onset, and no evidence of other tick-borne pathogens were found. The most common symptoms in the patients assessed were headache and fever. Other symptoms included coma, fatigue, depression, poor appetite, nausea, myalgia or arthralgia, and a rash. The symptoms resolved after six-to-eight days of treatment, and neither permanent clinical complications nor death occurred [15]. The ALSV has mostly been detected in *Ixodes ricinus* and *I. persulcatus* ticks [12] and is currently considered a tick-borne arbovirus. Later, the ALSV was detected in sheep and cattle in ALSV-endemic areas, which revealed its potential veterinary importance [23,24].

The ALSV has been detected in Eurasia, including France [25], Germany [24], Finland [19], Switzerland [20], China [15], and several regions across Russia [12,14,26]. In Russia alone, the ALSV is circulating across territories where more than ten million people live. Currently, no systematic ALSV surveillance exists in Russia, so the ALSV might be more widely distributed than the data demonstrate [12]. Various tick-borne pathogens such as the TBEV, Omsk haemorrhagic fever virus (OHFV), Powassan virus (POWV), and Louping ill virus (LIV) are circulating in Russia [5,26]. Recently, another member of the JMV group, the YGTV, was detected in different tick species in the territory where the ALSV was circulating [26]. The TBEV was also found in the same location as were the ALSV and YGTV, which indicates the possibility of a coinfection between classical members of the genus *Orthoflavivirus* and the JMV group [26]. The clinical manifestation of the ALSV is similar to that of the TBEV [15], which makes differentiating between the two viruses in the sympatric areas important for patient diagnosis, prognosis, and treatment.

Considering all the above-mentioned factors, a tool that can quickly and specifically detect the ALSV is required. The current approaches to ALSV detection in ticks mostly include RT-PCR assays, followed by result confirmation with sequencing [12,26,27]. Previously, we used RT-PCR assays targeting VP1a putative envelope protein [12,19,26]. Cai and co-authors used a nested RT-PCR system targeting the NS3 gene [27], while Kuivanen and co-authors used a RT-PCR system targeting the NS5 gene. Sometimes, high-throughput sequencing is used [19,27].

In two studies, serological screening based on the purified VP2 protein, as well as an RT-qPCR, was used. In both cases, serological assays based on the putative capsid protein VP2 of the ALSV, and SYBR RT-qPCR systems were used [23,24]. However, Ebert and co-authors RT-qPCRtargeted a 334 nt region of the NS5 gene of ALSV [24], while Wang and co-authors system targeted a region within the NS3 protein [23].

However, such assays were never proven to not provide false-positive results with a wide array of co-circulating viruses. With this study, we present a sensitive and specific RT-qPCR assay that can be used to detect viruses both in ticks and human serum.

## 2. Materials and Methods

### 2.1. Virus-Containing Materials, Serum and Ticks

The JMV group members, the ALSV strains Miass519 and Miass527 [14], and the YGTV strains Plast15-T22438 and Bredy15-T22181 [26] described previously were used for the current study. Several members of the genus *Orthoflavivirus* from the institute’s collection were used: the TBEV (strains Sofjin, EK-328, and Absettarov of the far-eastern, Siberian, and European subtype, respectively), LIV strain S1, POWV strain Pow-24, West Nile virus (WNV) strain SHUA-3, Japanese encephalitis virus (JEV) strain Gagar, and dengue-4 strain Cambodia. Kemerovo virus (KEMV) strain 21/10 (*Sedoreoviridae*, *Orbivirus*) from the institute’s collection was used. The full virus list, the system where the virus was replicated, and the amount of virus in plaque-forming units per mL (where possible) are presented in Table 1.

The poliovirus strain Sabin (10^4^ copies/μL) from the institute’s collection was used as the internal control for this study (see below).

To test the specificity of the assay, pools of the various species of the field-collected ticks were used. The tick species, pool composition, collection sites and collection dates are presented in Table 2. Before RNA isolation, the ticks were homogenised using TissueLyser II (QIAGEN, Germany) in a 0.9% saline solution (FSASI Chumakov FSC R&D IBP RAS, Moscow, Russia). The volume of the solution added depended on the tick species and the number of ticks in the pool; for each *Ixodes* spp. and *Haemaphysalis* spp. tick, 150 µL of the solution was added, and for each *Dermacentor* spp. tick, 200 µL of the solution was added.

Additionally, ten negative human serum samples received previously during clinical trials of the Tick-E-Vac vaccine [30], and a sheep serum (Gibco, New Zealand) were used for this study.

### 2.2. RNA Isolation

The total RNA was isolated from the samples using the TRI reagent LS (Sigma-Aldrich, St. Louis, MO, USA) in accordance with the manufacturer’s instructions. This method is based on the single-step method of phenol-chloroform extraction [31]. Briefly, 375 μL of the TRI reagent LS was added to 125 μL of the sample, and then 100 μL of chloroform was added. After that, the mix was shaken by hand for 2 min, left at room temperature for 15 min, and centrifuged at 12,000× *g* for 15 min by using the Eppendorf 5424 centrifuge (Eppendorf, Hamburg, Germany). After that, the water phase (on top) was transferred into the fresh tube with the addition of 250 μL of water; the tube was gently mixed, left at room temperature for 10 min, and centrifuged at 12,000× *g* for 8 min by using the Eppendorf 5424 centrifuge (Eppendorf). After that, the supernatant was discarded, 1 mL of 80% ethanol was added, and the samples were centrifuged at 7500× *g* for 5 min. Then, the ethanol was discarded from the mix and the precipitate was dried in the thermostat at 37 °C for 15 min. After the procedure, the RNA was dissolved in the water and was either instantaneously used for the qPCR or, if the porcine embryo kidney cell line’s RNA was being prepared, stored at −70 °C (Section 2.4).

### 2.3. Preparation of the Standard RNA Samples

The standard RNA samples were prepared as follows. First, a 1381 nt fragment of segment 2 of strain Miass527 was amplified with oligonucleotides (Table 3), with the T7 promoter merging with the forward primer. The obtained PCR product was gel-purified using the QIAGEN gel extraction kit (QIAGEN, Hilden, Germany). The purified PCR products were used for in vitro transcription using T7 RNA polymerase (Sibenzyme, Novosibirsk, Russia) in accordance with the manufacturer’s instructions. The obtained RNA was purified in a sucrose density gradient (5–20%) by using Optima L-90K Ultracentrifuge with an SW-40 rotor at 40,000 rpm for 4 h at 4 °C. The resultant density gradient fractions were screened for their RNA amount by using agarose gel electrophoresis, and the RNA was precipitated from the fraction with the highest amount of RNA as described in Section 2.2.

The amount of pure RNA obtained was measured using NanoDrop One^C^ (ThermoFischer Scientific, Madison, WI, USA). The RNA molar mass was manually calculated to determine the RNA fragment starting from the BHT7 promoter start base of the BHT7_Miass_VP1a_F3 oligonucleotide (Table 3) and ending at the Miass_gly_2R oligonucleotide. Using Equation (1), the RNA molecule quantity was calculated, ten-fold dilutions of the RNA in the water were prepared, and the RNA was stored at −70 °C for downstream applications.

RNA concentration (copies/μL) = (RNA concentration(g/μL) × N_A_)/RNA molar mass (1)

### 2.4. Preparation of Porcine Embryo Kidney Cell Line Total RNA

The porcine embryo kidney cell line’s total RNA was used to normalise the amount of total RNA in each sample during reverse transcription and qPCR. To isolate the total RNA of the porcine embryo kidney (PEK) cell line, 750 μL of the TRI reagent LS (Sigma-Aldrich, St. Louis, MO, USA) was added to the one-day PEK cells’ monolayer that was growing on the 25 cm^2^ cell culture flask (Corning, New York, NY, USA), with the cell supernatant being discarded before the procedure. The TRI reagent LS was then used to lyse the PEK cells, and the obtained mixture was used to proceed with the RNA isolation protocol described in Section 2.2.

The purified RNA was dissolved in 50 μL of water, the RNA concentration was measured by using NanoDrop One^C^ (ThermoFischer Scientific, Madison, WI, USA), and the RNA was subsequently aliquoted to 250 ng/μL and stored at −70 °C.

### 2.5. Reverse Transcription and qPCR

Prior to the RNA isolation procedure followed for qPCR, 1 μg of the RNA of the PEK cells (see Section 2.4) was added to each sample to normalise the amount of RNA between the samples. Additionally, 2 μL of the poliovirus strain Sabin (10^4^ copies/μL) was added to each sample as an internal control. The total RNA was isolated from the samples by using the TRI reagent LS (Sigma-Aldrich, St. Louis, MO, USA) in accordance with the manufacturer’s instructions (see above).

Reverse transcription was conducted on the total RNA by using MMLV Reverse Transcriptase (Evrogen JSC, Moscow, Russia) immediately after RNA isolation. Briefly, the RNA was dissolved in 9 μL of water, and 2 μL of Miass_gly_3R (5 pmol/μL) oligonucleotide was added (Table 4). The mix was incubated for 2 min at 70 °C and then placed on ice for 2 min. After that, 4 μL of the 5× reaction buffer (Evrogen JSC, Moscow, Russia), containing 280 mM Tris-HCl, at pH 8.7, 375 mM KCl, 30 mM MgCl_2_, 2 μL of DTT (Evrogen JSC, Moscow, Russia), 2 μL of 10 mM dNTP (Evrogen JSC, Moscow, Russia), and 1 μL of MMLV Revertase (Evrogen JSC, Moscow, Russia) was added to the reaction mix, and the mix was incubated at 42 °C for 1 h. Reverse transcription for the internal control sample was carried out using the same approach, with a specific PVR1 oligonucleotide being used (Table S1).

The qPCR was performed using the R-412 qPCR reaction kit (Syntol, Moscow, Russia) in accordance with the manufacturer’s instructions. Briefly, a mix containing 2.5 μL of 2.5 mM dNTP, 2.5 μL of the 10× buffer, 2.5 μL of 25 mM MgCl_2_, 2 μL of forward and reverse oligonucleotides, 1 μL of a qPCR fluorescent probe, 0.25 μL of SynTaq polymerase, and 10.25 μL of H_2_O was prepared (all the reagents were supplied by Syntol, Moscow, Russia). Then, 2 μL of the sample was added and the mixes were placed in C1000 Thermal Cycler (Bio-Rad, Hercules, CA, USA). Fluorescence detection was conducted using CFX96 Real-Time System (Bio-Rad, Hercules, CA, USA). For ALSV detection, the Miass_gly_3F and Miass_gly_3R oligonucleotides with a Miass_gly3_PROBE fluorescent probe (Table 4) were used. For the internal poliovirus control qPCR, the PVL1 and PVR1 oligonucleotides with a PVP1 fluorescent probe were used (Table S1). The exact amplification cycles for the ALSV and internal poliovirus control are presented in Table S3.

The obtained amplification data were analysed using Bio-Rad CFX Manager v.3.1 (Bio-Rad, Hercules, CA, USA); the “Single Threshold” Cq Determination mode, “Baseline subtracted Curve Fit”, and “Apply Fluorescence Drift Correction” baseline settings were used, and the other settings were used as a default.

For standard RNA samples, the mean quantification cycle was calculated, using Microsoft Excel 2010’s “AVERAGE“ function. The confidence of the mean value was calculated using a Student’s t distribution with Microsoft Excel 2010 (“CONFIDENCE.T” function; 95% confidence level). Results were presented in the “Mean ± confidence” format.

### 2.6. Virus Detection in the Serum Experiments

To test the ability of the qPCR system to detect the ALSV in sera, serum samples spiked with the ALSV were prepared. To conduct this, 25 μL of the ALSV strain Miass519 (9.7 × 10^7^ RNA copies/μL) were added to 100 μL of the serum sample that previously tested negative for the ALSV. Two separate experiments were conducted: one using human serum and another using sheep serum. The obtained spiked samples were then immediately used for RNA isolation followed by the qPCR.

## 3. Results

In previous studies, a Miass_gly_3F/Miass_gly_3R oligonucleotide pair (Table 4) was first designed to detect the ALSV strain Miass527 in the IRE/CTVM19 cell culture [14]. The pair targeted the VP1a putative envelope protein [11] and amplified a sequence that was 333 nt in length. Subsequently, this pair showed strong results in the screening of ticks for the presence of the ALSV, which allowed the discovery of more than 40 different ALSV isolates in ticks from various parts of Russia [12,14,26]. With this study, we further enhanced the detection system by designing the oligonucleotide fluorescent probe Miass_gly3_PROBE (Table 4, Figure S2). This allows for a less expensive and labour-intensive system while preserving its specificity and sensitivity, and it also allows for the ALSV in the sample to be quantified. Additionally, the system was supplemented with a poliovirus internal control qPCR to prevent false-negative results due to mistakes during the RNA isolation and reverse transcription procedures.

We tested the sensitivity of the assay by preparing standard RNA samples of the fragment of segment 2 (Section 2.3) and using ten-fold RNA dilutions in a qPCR. Examples of the amplification curves produced by standard RNA samples are shown in Figure S3A and S3B. In order to clearly establish the copy number limit, an additional experiment was performed with 8 × 10^3^–2 × 10^3^ copies of RNA. However, no amplification was observed in these samples (Table 5). Overall, the lowest amount of RNA that showed amplification was 10^4^.

**Figure 1:**
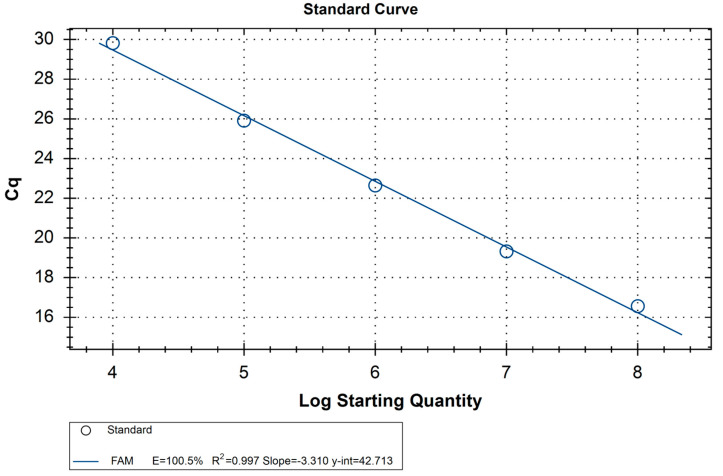
Amplification curve of the standard RNA of a known amount.

The quantification cycle (Cq) for the standard samples ranged from 29.5 (10^4^ RNA copies) to 16.1 (10^8^ RNA copies). The linear dependence between the amount of RNA copies and Cq had an R^2^ value of 0.997 (Table 5, Figure 1). This indicates that this system may be used in conjunction with standards to calculate the virus load in the sample.

Because 10^4^ copies was the lowest amount of RNA copies that could provide a signal, in further work, a sample was considered positive if the calculated amount of the virus cDNA in the sample was higher than 10^4^ (Cq of 29.7). Otherwise, the amplification was considered nonspecific.

We tested the specificity of the qPCR system with several possible targets. All the samples mentioned below were tested to amplify the internal control poliovirus RNA that was added to the sample before isolation. First, we tested the system with two ALSV strains: Miass519 and Miass527. The qPCR system was able to successfully detect both viruses in the samples (Table 5). To test the ability of the system to detect the ALSV in the serum samples, we used human and sheep sera. Both sera were tested with and without spiking them with 25 μL of the ALSV strain Miass519. We were able to successfully detect the ALSV in the spiked sera (Table 5). In ten samples of nonspiked human sera, as well as in the nonspiked sheep serum, no amplification was detected. Examples of the amplification curves produced by positive and negative samples are shown in Figure S3C,D.

Additionally, we used a panel of the tick-borne orthoflaviviruses, including the three most common genotypes of the TBEV, POWV, OHFV, and LIV. We report no detectable amplification of those viruses by our system.

The second panel included several other viruses, including highly relevant mosquito-borne orthoflaviviruses: the Japanese encephalitis virus, dengue 4 virus, and West Nile virus. Additionally, we used two strains of the YGTV, which is a close relative of the ALSV and Kemerovo virus, a tick-borne orbivirus that was isolated in Russia from *I. persulcatus* ticks. We report no detectable amplification of those viruses by our system. We detected no amplification of the viruses replicated in the PEK cells, Vero cells, and mouse brain (Table 1). Because these samples also contained host RNA and we detected no amplification, we can conclude that no amplification would have been detected in the virus-free PEK cells, Vero cells, or mouse brain as well.

One of the most predominant procedures used in epidemiological studies is the testing of collected ticks for the presence of a virus. Thus, we tested our system’s specificity with two of the most common tick species in Russia, *Ixodes ricinus* and *I. persulcatus*, as well as several other ticks that can be found in Russian territories: *Dermacentor reticulatus, D. marginatus, Haemaphysalis concinna*, and *H. japonica*. The ticks used in this experiment were studied in pools of two to six specimens in the sample and were previously tested for the absence of orthoflaviviruses, the ALSV, and the YGTV using an RT-PCR. No detectable amplification was detected in all the tick samples.

Overall, only the samples containing the ALSV samples produced an amplification signal higher than the 10^4^ copy threshold that we established earlier for this system (Table 5). This shows that the qPCR system, together with standard RNA, did not provide false positive reactions for any systems tested in this study. At the same time, the qPCR system successfully detected the ALSV in the two virus strains obtained during laboratory collection, as well as in the sera spiked with the ALSV.

## 4. Discussion

The ALSV is spread across Europe [19,20,25], China [15,23], and throughout several regions in Russia [12,14,26]. Throughout the Russian territory, the ALSV is actively cocirculating with the TBEV in the Republic of Karelia [26,32], Chelyabinsk Region [12,26], and the Republic of Tuva [12,33], which is leading to the possibility of coinfections. Currently, little is known about the biology of the ALSV, as both the host range and pathogenic potential of the virus are not precisely known [15]. To estimate the epidemiological importance of the ALSV, we need to accurately measure the presence of the virus in ticks, cattle, and humans.

This situation makes the development of a system that can quickly and easily detect the ALSV necessary. Currently, as shown by the results of previous studies, ALSV presence is usually analysed via a RT-PCR, followed by Sanger sequencing [12,26,27]. For one study, immunofluorescence assays and an SYBR Green RT-qPCR were used. The prevalence of the ALSV found using the ELISA method was much lower than that found when using a RT-qPCR [23]. Researchers think that this situation is the result of collecting serum from young animals (of less than one year old) in the beginning of the epidemic season of ticks [23]. However, no data on the specificity and sensitivity of the abovementioned assays exist, so this situation could also be the result of the higher sensitivity of the RT-qPCR or its low specificity. Overall, this situation highlights the necessity for assays with proven specificity, especially if research is conducted in cocirculation areas.

Additionally, many of the RT-PCR and RT-qPCR systems available today mostly use oligonucleotides that are specific to the virus polymerase or helicase [15,23,24]. These proteins are very conservative with a high homology not only among the JMV group but the genus *Orthoflavivirus* as well. This leads to a situation where cross-reactions within the JMV group and genus *Orthoflavivirus* are possible. For example, in some cases, genus *Orthoflavivirus* polymerase-specific oligonucleotides [34], which have very limited homology to the JMV group, can amplify the ALSV and YGTV [14]. For this study, we used a different approach using oligonucleotides that target the VP1a protein, with very limited homology to the genus *Orthoflavivirus* and lower homology within the JMV group.

During testing, our qPCR system amplified two different ALSV strains and did not amplify the YGTV, the three most common TBEV genotypes, or several other orthoflaviviruses (both tick and mosquito borne) that can cause coinfection in Russia. Our system also produced no amplification signals in ALSV-free *I. ricinus, I. persulcatus, D. reticulatus, D. marginatus, H. concinna*, and *H. japonica* or in ALSV-free sheep and human sera. These data highlight that this system can be used for the differential detection of the ALSV in various organisms. JMV group members are often isolated in *I. Ricinus*-and *Hyalomma anatolicum*-derived cell cultures [12,14,26], with many more tick-derived cell cultures available [28,29]. We can assume that the qPCR system would also provide no nonspecific amplification tick cell lines derived from *I. ricinus, I. persulcatus, D. reticulatus, D. marginatus, H. concinna*, and *H. japonica* ticks because the system reported no amplification signal in the ticks that the cultures were derived from.

Overall, the qPCR system that we present can detect ALSV in ticks, the blood serum of humans and animals, and a variety of common cell cultures, which makes it a useful tool for human diagnostics, epidemiological studies of ticks, and laboratory experiments involving the ALSV. One additional benefit of our system is that with an amplicon length of 333 nt, Sanger sequencing can be used to confirm a positive result if necessary. Our system was designed to have a poliovirus internal control qPCR, as well as the RNA of PEK cells to normalise the RNA amount in the samples. The system was designed this way based on the availability of these exact reagents in our lab. However, they can be easily replaced with RNA from other sources if needed because the qPCR assay showed high specificity.

For the TBEV, virus loads in the serum were not associated with the patients’ clinical parameters, such as the duration of the first phase of the disease, the duration of the asymptomatic interval, TBE severity, and clinical presentation [35]. However, higher virus loads in the plasma predicted the development of WNV infection symptoms [36,37]. Currently, no data on the importance of the virus load for the ALSV infection course exist, but that data can be obtained by using our system.

The TBEV, as a member of the genus *Orthoflavivirus*, shares some homology with JMV members [9,10,11], particularly with the ALSV. The TBEV testing systems were designed before the JMV group was discovered, so the specificity of those systems when detecting the ALSV has not been tested yet. According to the sanitary rules in Russia, when a potential patient brings a tick to a laboratory, if the tick is positive for TBEV RNA, a specific immunoglobulin is administered [5]. However, in the case of the false-positive identification of the ALSV as the TBEV, an immunoglobulin prescription would be useless.

Considering the cocirculation and homology of ALSV and TBEV, to obtain accurate epidemiological data on both the ALSV and TBEV, reliable systems that can detect both viruses are needed. We describe a specific system for ALSV detection; however, further research is required to test the specificity of and, if necessary, enhance the existing TBEV-detection systems.

## Figures and Tables

**Table 1:** Viruses used for the testing of the qPCR system.

Virus	Strain	Virus Amount, PFU/mL	Virus Origin
ALSV	Miass519	+ ^1^	HAE/CTVM8 ^2^ cells
ALSV	Miass527	+	IRE/CTVM19 ^3^ cells
YGTV	Plast15-T22438	+	HAE/CTVM8 cells
YGTV	Bredy15-T22181	+	HAE/CTVM8 cells
TBEV	Sofjin	6.7	Mouse brain
TBEV	EK-328	7.1	PEK ^4^ cells
TBEV	Absettarov	9.5	Mouse brain
OHFV	Nikitina	7.6	PEK cells
WNV	SHUA-3	7.7	Vero cells
LIV	S1	6.2	Mouse brain
POWV	Pow-24	7.6	PEK cells
JEV	Gagar	8.1	PEK cells
Dengue-4	Cambodia	+	Mouse brain
KEMV	21/10	7.1	PEK cells

^1^ Virus amount was unknown; the presence of the virus was confirmed via virus-specific PCR (Figure S1). ^2^ Cell line originating from *Hyalomma anatolicum* ticks [28]. ^3^ Cell line originating from *Ixodes ricinus* ticks [29]. ^4^ Porcine embryo kidney cell line.

**Table 2:** Ticks used in the work.

Tick Species	Number of Ticks in a Pool	Collection Site	Collection Date
*Ixodes ricinus*	6 ♂	Russia, Kaliningrad Region	2017
*Ixodes ricinus*	5 ♀	Russia, Kaliningrad Region	2017
*Ixodes persulatus*	5 ♀	Russia, Primorsky Territory	2021
*Dermacentor retuculatus*	3 ♀	Russia, Chelyabinsk Region	2015
*Dermacentor marginatus*	4 ♀	Russia, Chelyabinsk Region	2015
*Haemaphysalis conccina*	2 ♂	Russia, Primorsky Territory	2021
*Haemaphysalis japonica*	2 ♀	Russia, Primorsky Territory	2021

**Table 3:** Oligonucleotides used for the preparation of the standard RNA samples.

Oligonucleotide	Sequence
BHT7_Miass_VP1a_F3	5′-ATGACTGGATCCTAATACGACTCACTATAG*GCTTGTAAAGCTAGCGACTGGA-3′
Miass_gly_2R	5′-AAAGCCTCATGGACGGTCTG-3′

* First base of the RNA transcript.

**Table 4:** Oligonucleotides used for ALSV-specific qPCR.

Oligonucleotide	Sequence	Location
Miass_gly_3F	5′-TGGATCAGCTCACACCACAC-3′	VP1a
Miass_gly_3R	5′-TCACCGTCACAGTGGAATGG-3′	VP1a
Miass_gly3_PROBE	(FAM)-TTGCGACCCCGTTGTCGTCG-(BHQ-1)	VP1a

**Table 5:** Samples where amplification was detected during qPCR.

Probe	Cq	Quantity	Detection Result
10^8^ Standard RNA	16.1 ± 0.8 *	10^8^	-
10^7^ Standard RNA	19.1 ± 0.8 *	10^7^	-
10^6^ Standard RNA	22.3 ± 0.8 *	10^6^	-
10^5^ Standard RNA	25.9 ± 1.1 *	10^5^	-
10^4^ Standard RNA	29.5 ± 1 *	10^4^	-
8 × 10^3^ Standard RNA	no amplification	8 × 10^3^	-
6 × 10^3^ Standard RNA	no amplification	6 × 10^3^	-
4 × 10^3^ Standard RNA	no amplification	4 × 10^3^	-
2 × 10^3^ Standard RNA	no amplification	2 × 10^3^	-
10^3^ Standard RNA	no amplification	10^3^	-
ALSV strain Miass519	15.8	9.7 × 10^7^	positive
ALSV strain Miass527	7.4	3.6 × 10^10^	positive
Human serum, spiked with ALSV strain Miass519	18.5	1.7 × 10^7^	positive
Sheep serum, spiked with ALSV strain Miass519	19	1.2 × 10^7^	positive

* Average Cq of five qPCR runs.

## Data Availability

The data presented in this study are available in the manuscript and Supplementary Materials. Raw qPCR data is available upon request.

## Supplementary Materials

The following supporting information can be downloaded at https://www.mdpi.com/article/10.3390/mps6050079/s1. Figure S1: Gel electrophoresis of Yanggou tick virus (YGTV) samples used in the work; Figure S2: An alignment of prototype strains of the Jingmenvirus (JMV) group members; Figure S3: Examples of the amplification curves produced via an Alongshan virus qPCR; Table S1: Oligonucleotides used for the RT-qPCR of the internal control Poliovirus; Table S2: Amplification cycle for the ALSV qPCR; Table S3: Amplification cycle for the Poliovirus qPCR.
