# Supplementary material for: Quantitative Polymerase Chain Reaction System for Alongshan Virus Detection
Source: Methods Protoc. 2023 Sep 4;6(5):79. doi: 10.3390/mps6050079 (PMC10514782; doi:10.3390/mps6050079)
Supplement: Supplementary file 1 [file mps-06-00079-s001.zip › mps-2515688-supplementary.pdf]

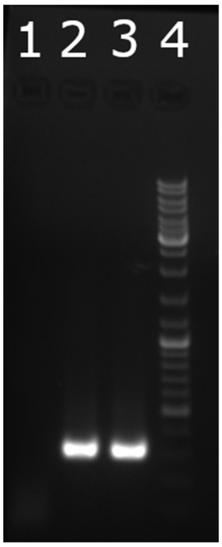

Figure S1. Gel electrophoresis of Yanggou tick virus (YGTV) samples used in the work. Lane 1 – negative control, lane 2 – YGTV strain Plast15-T22438; lane 3 – YGTV strain Bredy15-T22181; lane 4 – GeneRuler DNA ladder Mix (ThermoFischer Scientific).

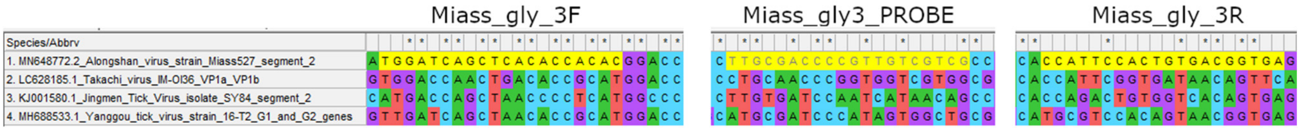

Figure S2. An alignment of prototype strains of the Jingmenvirus (JMV) group members. Regions of the discussed oligonucleotides are marked above and highlighted in yellow.

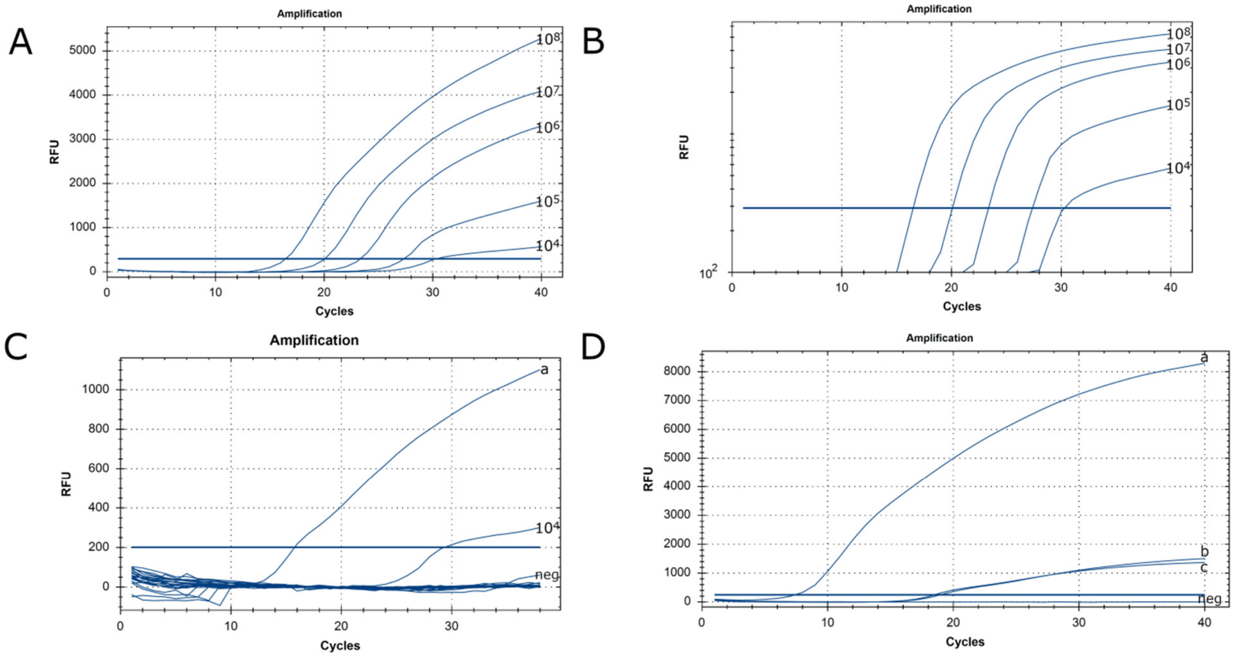

Figure S3. Examples of the amplification curves produced via an Alongshan virus qPCR. A. Amplification curves of the standard RNA samples. B. Amplification curves of the standard RNA samples (logarithmic fluorescence scale). C. Amplification curve of the strain Miass519 (a), compared with 10^4 standard RNA sample and multiple non-amplifying samples (neg). D. Amplification curve of the strain Miass527 (a), as well as 100 µl sera samples (b, c) spiked with 25 µl of the ALSV strain Miass519 (9,7*10^7 RNA copies/µl), and multiple non-amplifying samples (neg).

Table S1. Oligonucleotides used for internal control Poliovirus RT-qPCR.

Oligonucleotide	Sequence
PVR1	5’-CGAACGTGATCCTGAGTGTT-3’
PVP1	(ROX)-TTGATTCATGAATTTCCTTCATTGGCA-(BHQ-2)
PVL1	5’- GGCAGACGAGAAATACCCAT- 3’

Table S2. Amplification cycle for ALSV qPCR.

**Amplification step**	**Instruction**
1	**Hold**, 95C, 3 min
2	**Hold**, 95C, 10 seconds
3	**Hold**, 58C, 30 seconds
4	**Plate read**
5	**Goto** 2, 39 times

Table S3. Amplification cycle for Poliovirus qPCR.

**Amplification**	**Instruction**

step	
1	**Hold**, 95C, 5 min
2	**Hold**, 95C, 15 seconds
3	**Hold**, 60C, 45 seconds
4	**Plate read**
5	**Goto** 2, 42 times
